# Topical formulation of sodium deoxycholate for submental lipolysis

**DOI:** 10.1111/srt.13293

**Published:** 2023-03-07

**Authors:** Hye Jeong Park, Ji Sung Jang, Chul Hwan Kim, Kyoung‐Hee Kim, Geewoo Nam

**Affiliations:** ^1^ SCAI Therapeutics Yuseong‐gu Daejeon Republic of Korea

To the Editor:

Changes in the submental region play a critical role in the facial manifestation of aging. A key component of this phenomenon, submental fat (SMF) or “double chin” is a condition associated with fat accumulation below the chin.^1^ Surgical procedures (e.g., submental liposuction), associated with various postoperative complications, were dominantly employed to treat SMF before 2015.^2^ For these reasons, FDA approval of an injectable composition containing deoxycholic acid (DCA) as the active pharmaceutical ingredient (Kybella) for the treatment of moderate‐to‐severe supraplatysmal SMF in 2015 was met with a surge of popularity. Introduction of Kybella led to a substantial diminution in the invasiveness of SMF reduction through subcutaneous injections. Recent studies, however, present the adverse effects from the injection of DCA‐based solutions in the form of marginal/pseudomarginal mandibular nerve injury, bruising, injection site alopecia, localized skin necrosis, and superficial hypoechoic lesions.^2^, ^3^, ^4^ Complications associated with SMF reduction using Kybella stem from painful repeated subcutaneous injections. Herein, we introduce a proprietary topical formulation of sodium deoxycholate (SCAI‐NaDC), developed by SCAI Therapeutics, capable of reducing SMF with minimal side effects.

Cellular studies demonstrated SCAI‐NaDC's ability to sufficiently lyse preadipocytes (3T3‐L1) at the following concentrations: 0.04%, 0.08%, and 0.1% (w/v) (Figure S1). Live cell imaging revealed that SCAI‐NaDC induced cellular membrane disruption in differentiated 3T3‐L1 adipocytes (Figure S2). SCAI‐NaDC's transdermal diffusion was investigated using a synthetic membrane, Strat‐M, in an Ussing chamber. SCAI‐NaDC (1%, w/v) yielded a membrane permeation of 50.4 ± 9.6 μg/cm^2^ after 72 h. Following these studies, clinical investigations were conducted to confirm the efficacy of SCAI‐NaDC in reducing SMF through topical administration.

Our 8‐week clinical study recruited a total of 21 female participants of approximately 47.9 years of age that satisfied its predetermined conditions to evaluate the reductive effects of SCAI‐NaDC on SMF. Participants were instructed to apply the SCAI‐NaDC product once a day over 8 weeks. Further details regarding cellular, ex vivo, and clinical experiments are discussed in the Supporting Information. All clinical photographs are presented in Figures S3 and S4.

A topical formulation of SCAI‐NaDC was prepared with an NaDC concentration of 1.0%. Our clinical studies demonstrated substantial reductions in SMF through two distinct visualization techniques measuring SMF area and volume. First, the area of the submental region was determined by taking and analyzing the photographs in pixels (Figure 1). Starting at 5272 pixels, topical application of SCAI‐NaDC led to a decrease in the submental area to 4894 and 4614 after 4 and 8 weeks, respectively. These changes correspond to 7.2% and 12.5% decrease in SMF.

**FIGURE 1:**
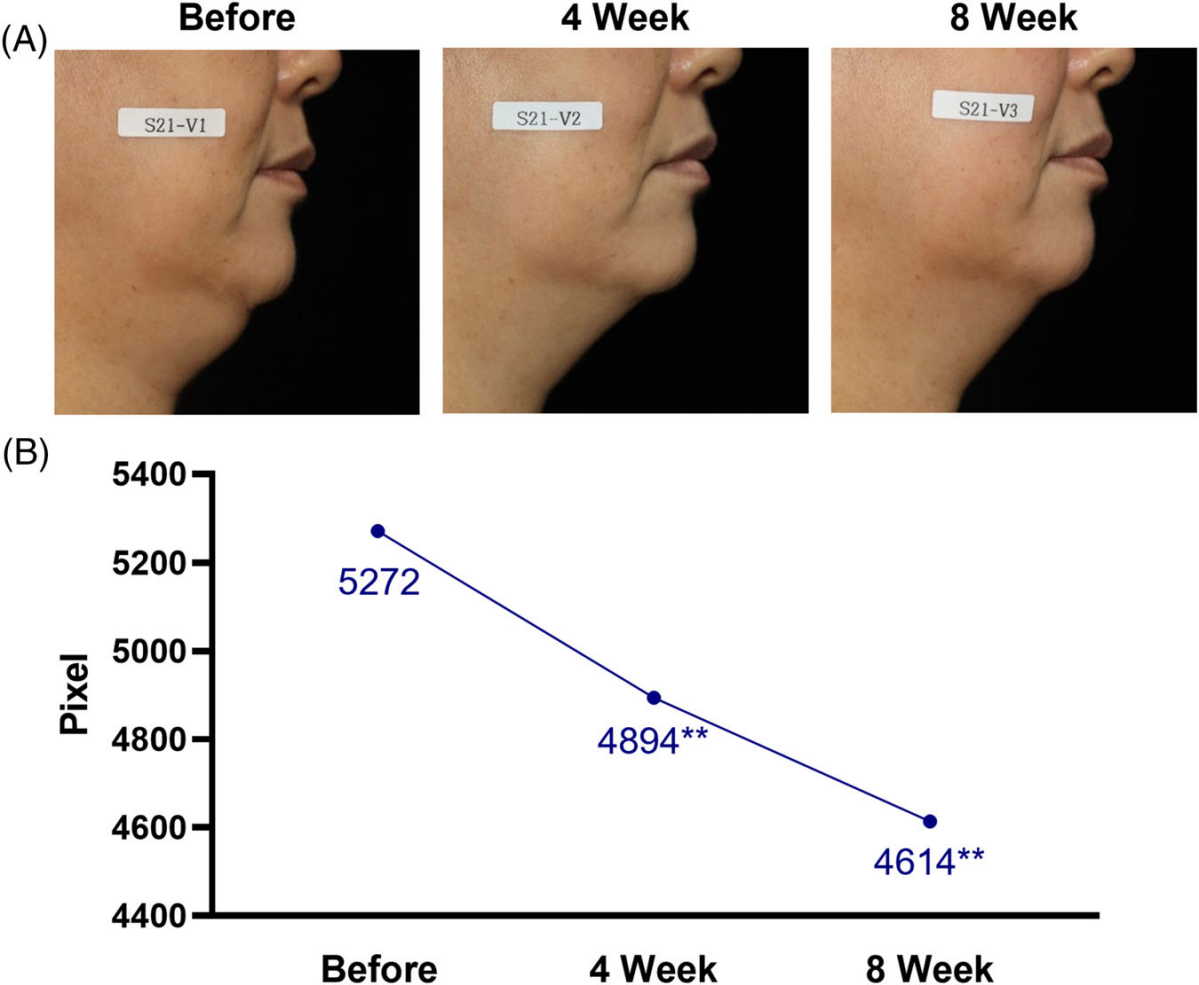
Clinical assessment of double chin reduction by area (pixel). (A) Representative images from the visual assessment of the submental area. The first image from the left was taken before the application of SCAI‐NaDC, the second and third images were obtained after 4 and 8 weeks of daily SCAI‐NaDC topical application. (B) Average submental fat (SMF) area was determined by ImagePro from 21 participants. **p < 0.05 by repeated measures ANOVA, post hoc Bonferroni correction.

A Vectra XT (Canfield Imaging Systems, USA) 3D imaging system was employed to determine the volume (mL) of the submental region (Figure 2). Starting from 10.0 mL, SMF volume was lowered to 9.1 and 8.5 mL after 4 and 8 weeks of SCAI‐NaDC application, respectively. These changes correspond to 9.2% and 15.0% reductions in SMF.

**FIGURE 2:**
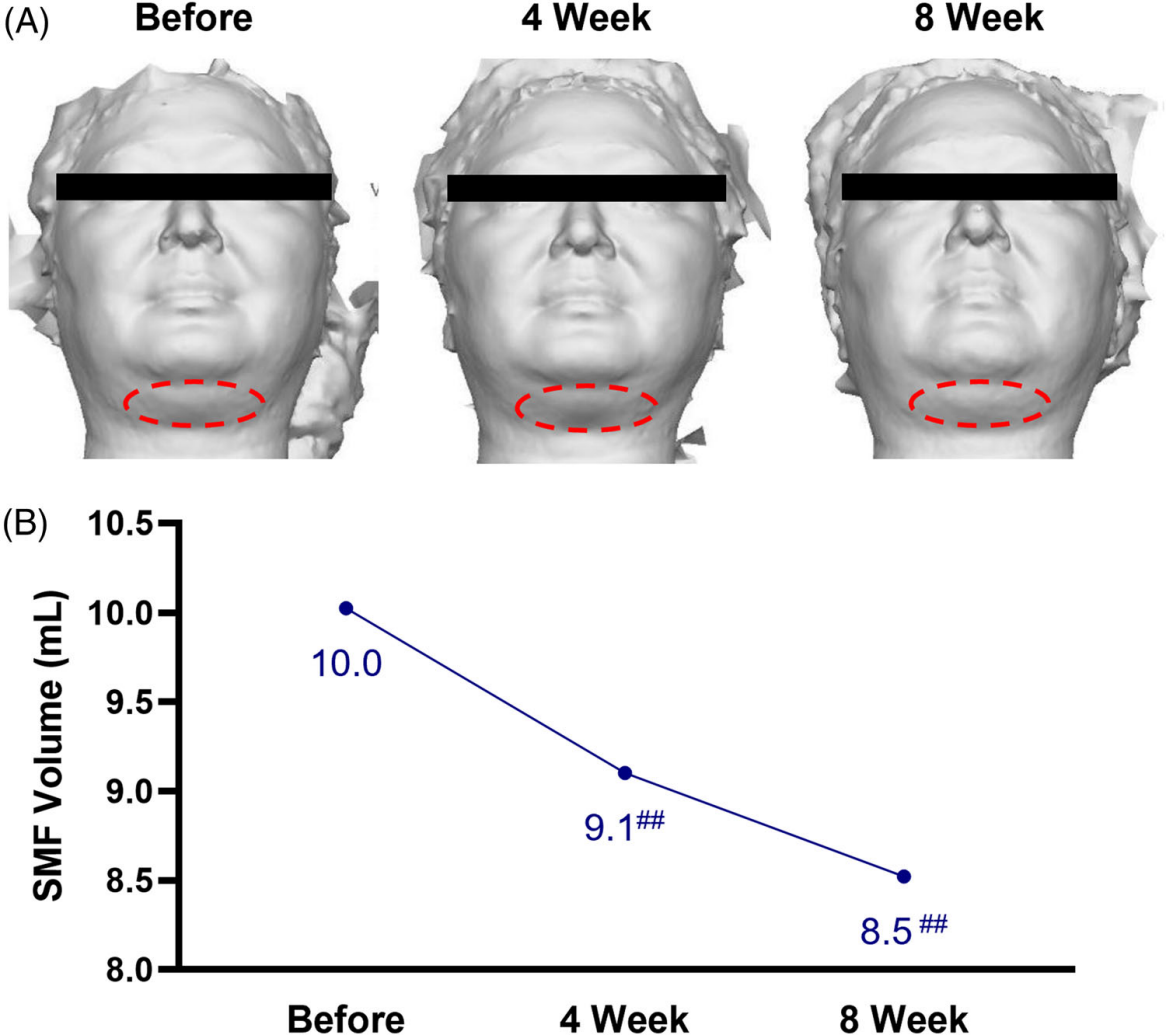
Clinical evaluation of the reductive effects of SCAI‐NaDC topical application on submental fat (SMF). (A) Representative images from the 3D evaluation of the submental region. Red highlights indicate the region of interest for numerical analysis. (B) Average SMF volume determined before and after 4 and 8 weeks of SCAI‐NaDC application. ^##^ *p* < 0.025 by Friedman test, post hoc Wilcoxon signed‐rank test with Bonferroni correction.

Overall, our interdisciplinary studies illustrate SCAI‐NaDC's ability to reduce SMF or “double chin” through topical administration, obviating the need for repeated subcutaneous injections associated with adverse effects. At the cellular level, SCAI‐NaDC effectively induced cytotoxicity in 3T3‐L1 cells through cellular membrane disruption. Ex vivo assessment of skin penetration showed that SCAI‐NaDC could potentially deliver NaDC to the SMF upon topical treatment. Clinical investigations demonstrated a substantial reduction in SMF area (12.5% in 8 weeks) and volume (15.0% in 8 weeks). These results denote the possibility of treating SMF by simply applying our topical formulation of NaDC. We hope to conduct further research to (i) establish a comprehensive understanding of SCAI‐NaDC and its ability to reduce SMF as a topical formulation, and (ii) develop a product making SMF reduction readily available with minimal pain and complications. No adverse effects were reported throughout the duration of the studies.

## ETHICS STATEMENT

Randomized clinical studies of NaDC were conducted with IRB approval (P2005‐1230) in accordance with the Code of Ethics based on the Helsinki Declaration, applicable human testing guidelines, and relevant regulations. The studies were conducted between May 2020 and August 2020 at PNK Skin Research Center in Seoul, South Korea. All recruited subjects have provided written informed consent.

## Supporting information

Supporting Information

## Data Availability

The data supporting this study are available from the corresponding author upon reasonable request.
