# Supplementary material for: Topical formulation of sodium deoxycholate for submental lipolysis
Source: Skin Res Technol. 2023 Mar 7;29(3):e13293. doi: 10.1111/srt.13293 (PMC10155785; doi:10.1111/srt.13293)
Supplement: Supplementary file 1 — Supporting Information [file SRT-29-e13293-s001.pdf]

Supporting Information for

Topical Formulation of Sodium Deoxycholate for Submental Lipolysis

Cellular Studies

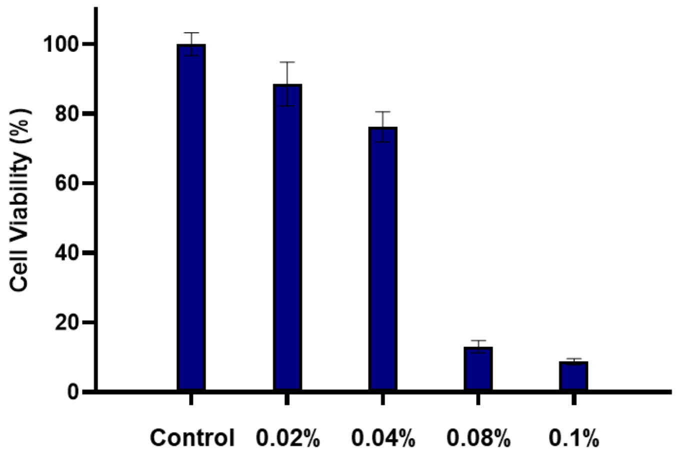

Figure S1. Cell viability assay evaluating the cytotoxicity of SCAI-NaDC against 3T3-L1 preadipocytes. Cells were treated with the compound at the following final concentrations: 0.02, 0.04, 0.08 and 0.1% (w/v). All experiments were performed in quadruplicates. Cell viability (%) was calculated relative to that of the control group.

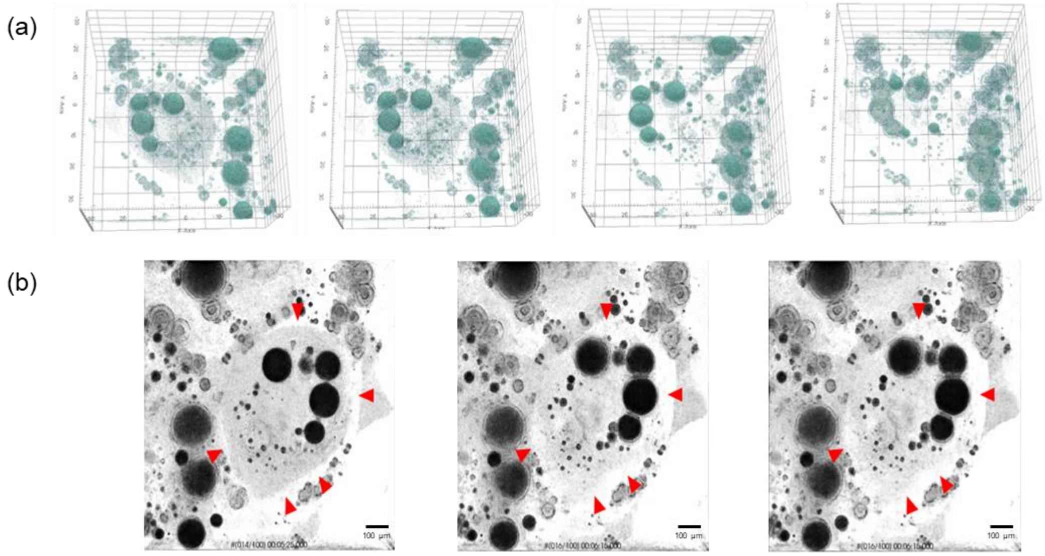

Figure S2. 3D and microscopic images temporally monitoring SCAI-NaDC-induced cellular membrane disruption in differentiated 3T3-L1 adipocytes. (a) 3D images showing the dissipation of intracellular lipid droplets in adipocytes. (b) microscopic images of adipocytes demonstrating the disruption of the cellular membrane. Cells were treated with SCAI-NaDC at a final concentration of 0.07% (w/v). Immediately following compound treatment, multipoint acquisition was used to monitor morphological changes in the adipocytes for 40 min in 25 sec intervals.

Materials and Methods

All reagents were purchased from commercial suppliers and used as received unless noted otherwise. NaDC (Sigma-Aldrich, MI, USA) was used to prepare SCAI-NaDC and as the control product for this study. Strat-M® membrane was purchased from Merck (Kenilworth, NJ, USA). 3T3-L1 preadipocytes was procured from Korea cell line bank (Seoul, Korea). Cell counting kit-8 was purchased from Dojindo (Rockville, MD, USA). FLUOstar Omega Microplate reader was purchased from BMG Labtech (Ortenberg, Germany). 3T3-L1 differentiation kit was purchased from Biovision (Waltham, MA, USA). 3D and 2D cellular images were taken with HT-2H microscope (Tomocube; Daejeon, Korea) accompanied by the corresponding imaging software (TomoStudio). HPLC analysis was performed using an e2695 (Waters; Milford, MA, USA) equipped with a C18 column. The SCAI-NaDC formulation was designed to concoct a easy to use and stable cream formulation based on previous experience to contain the following substances: distilled water, butylene glycol, propanediol, hydrogenated rice bran oil, caprylic/capric triglyceride, pentylene glycol, sodium deoxycholate, dimethicone, hydrogenated lecithin, hydroxyethyl acrylate/sodium acryloyldimethyl taurate copolymer, and carbomer.

Clinical Study

A total of 21 female subjects of approximately 47.9 years of age that satisfied the purposes of the study were recruited in the 8-week trial. The study comparatively evaluated the effects of SCAI-NaDC and Native NaDC on SMF reduction. The subjects were selected based on (i) visual appearance of double chin, and (ii) lack of any acute or chronic conditions including skin disorders. The following factors were considered in subject exclusion: history of medical treatments for submental fat reduction including liposuction, surgery, and lipolytic agent administration, radiofrequency treatment, laser, chemical, filler injection treatments in the chin and neck area within 1 year, botulinum toxin injection treatment in the chin and neck region within a 24-week period, pregnancy, psychological disorders, administration of immunosuppressants within 3 months, administration of systemic steroid or radiation therapy within 1 month, presence of lesions that may hinder measurements, atopic dermatitis, extreme skin sensitivity or allergies to cosmetics, pharmaceutics, and light, chemical peel, or skin treatment within 3 months. Randomized clinical studies of SCAI-NaDC was conducted with IRB approval (P2005-1230) in accordance with the Code of Ethics based on the Helsinki Declaration, applicable human testing guidelines, and relevant regulations. The clinical cosmetic efficacy studies were conducted between May 2020 and July 2020 at PNK Skin Research Center in Seoul, Korea. All recruited subjects have provided written informed consent. The participants were trained to clean and dry their hands before the application of equivalent amounts of each solution (single pump). The products, stored at room temperature in the dark, were administered daily for eight weeks. Throughout the study, participants were instructed to avoid the use of cosmetic products containing any active ingredients (e.g., whitening, anti-wrinkle). Subjects made 3 visits for clinical evaluation: before product application, after 4 weeks of product application, and 8 weeks of product application. Participants were trained to immediately report any abnormal and adverse effects following product application.

Submental Fat Reduction (Double Chin Lifting) Evaluation

To ensure the accurate assessment of SMF reduction, all subjects were accommodated in a controlled environment at 20-24 °C and 40-60% humidity for 30 min before instrumental analysis. Repeated measurements were taken in the same area of the face each time. Assessment of double chin lifting was conducted by measuring the area and volume through two distinct visual evaluation techniques. First, the double chin area was measured by repeatedly taking photographs of the region of interest using a DSLR camera (Canon EOS 800D, Japan) and analyzing said images with Image Pro® plus (Media Cybernetics, USA). The area of SMF region was represented in pixels. Reduction in the pixel values denote the reduction in the submental region and reduced SMF. A Vectra XT (Canfield Imaging Systems, USA) 3D imaging system was employed to image the submental region and determine the volume (mL) corresponding to SMF. Statistical analyses of the obtained data were performed via SPSS 19.0. Data sets with more than 3 data points were analyzed by repeated measures ANOVA, post hoc Bonferroni. Nonparametric methods used the Friedman test, post hoc Wilcoxon signed-rank test with Bonferroni correction. Comparative analysis between the control and experimental groups were performed regarding the temporal differential in SMF parameters using the Mann Whitney U test.

Artificial Membrane Penetration

To evaluate the skin penetration of SCAI-NaDC, Strat-M® membranes were installed in custom-designed Ussing chambers. The skin penetrations were conducted over a 72-hour period at 32 °C in an incubator. Quantification of the SCAI-NaDC was conducted via HPLC. Analytical conditions were as follows: Column - RP C18 (215x4.6); Mobile phase - Water:ACN:85 % H_3PO_4 (50:50:0.1); Flow rate - 1.0 mL/min; Injection volume - 10 µL; Run time - 17 min; Wavelength - 195 nm; Sample Temp. - 30 °C; Column Temp. - 25 °C.Experiments were performed in triplicate.

Cell Viability Test

3T3-L1 cell line was maintained in a high glucose DMEM media containing 10% bovine calf serum and 100 µg/ml streptomycin. The 3T3-L1 cells were grown and maintained at 37 °C in a humidified atmosphere with 5% CO_2. 3T3-L1 preadipocytes (P7) were seeded in a 96-well plate (5,000 cells/well) in 100 µL of media and incubated overnight. To these cells SCAI-NaDC were treated at final concentrations of 0.02, 0.04, 0.08 and 0.1% for 4 h. Following compound treatment, 10 µL of the cell counting kit-8 (CCK-8) solution was added to each well and incubated for 2 h. Absorbance at 450 nm was measured using a FLUOstar Omega microplate reader (BMG Labtech; Ortenberg, Germany). All experiments were performed four times and the average was calculated and analyzed to determine the cell viability of each group.

Cellular Membrane Imaging

To visually monitor the disruption of the cellular membrane induced by SCAI-NaDC, 3T3-L1 preadipocytes were differentiated to adipocytes using the Biovision 3T3-L1 differentiation kit. Upon confirmation of adipocyte differentiation, SCAI-NaDC was treated to the cells at a

concentration of 0.07%. Then, 3D images of the adipocytes were obtained using a HT-2H microscope (Tomocube; Daejeon, Korea) accompanied by the corresponding imaging software (TomoStudio). Imaging occurred at 25 second intervals for 40 min.

Safety Evaluation

Safety evaluations of the topical formulation SCAI-NaDC were conducted in two ways: (i) periodic self-evaluation by the clinical subjects and (ii) comprehensive evaluation conducted by a dermatology specialist before and after 4 and 8 weeks of product application. Throughout the duration of these studies, no noticeable dermatological changes or adverse effects were reported, denoting the clinical safety of our topical NaDC formulation.

Clinical Photographs

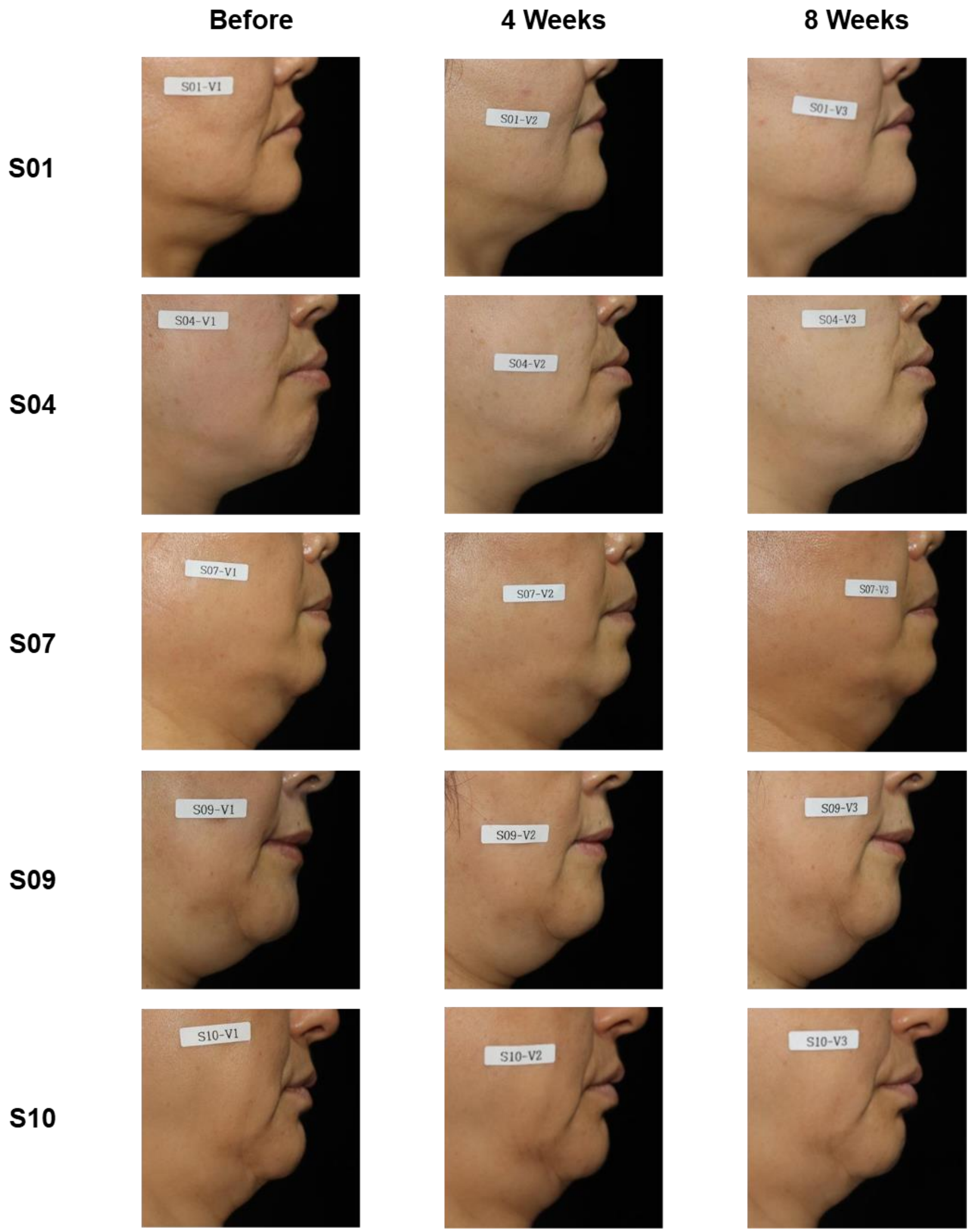

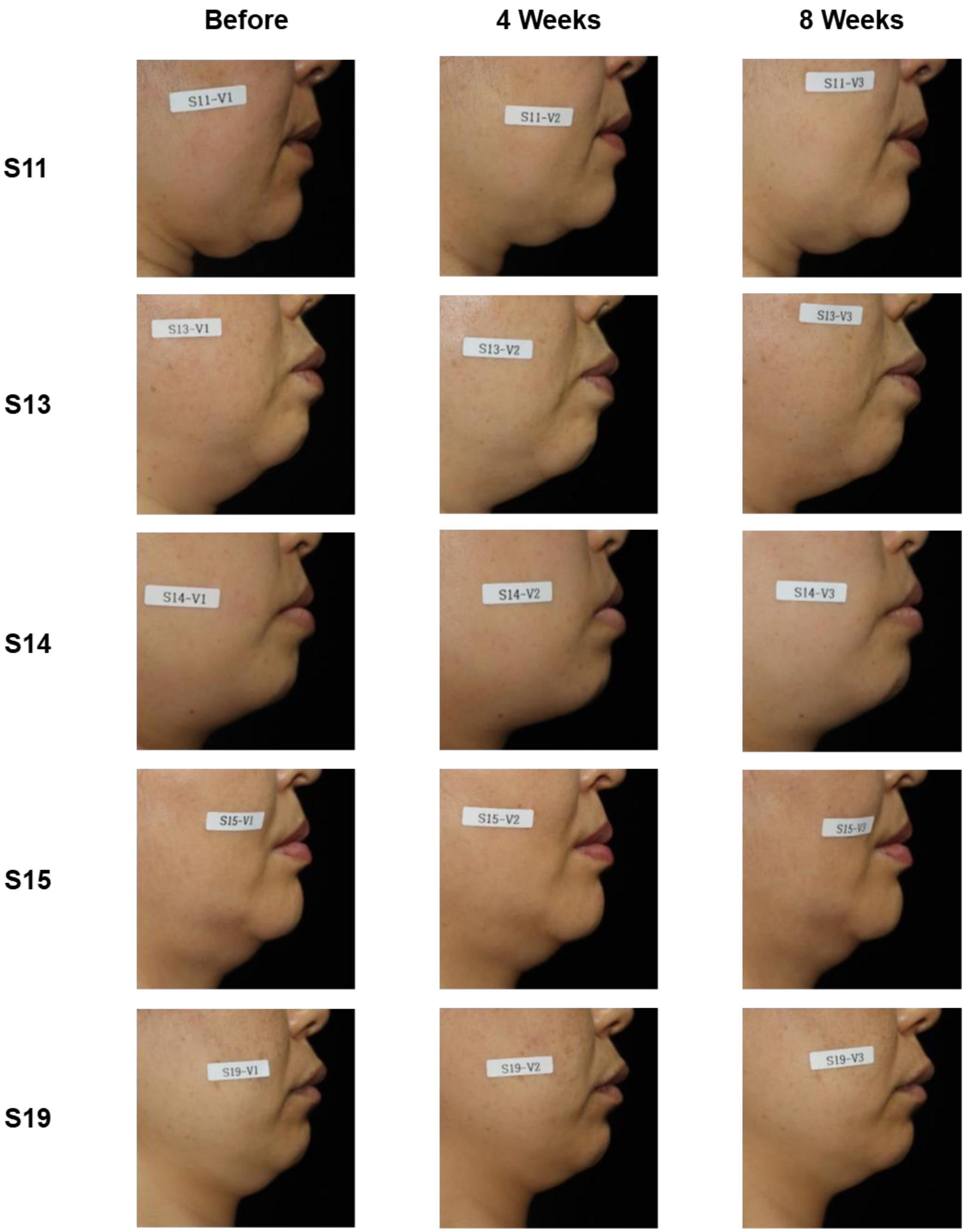
Before
4 Weeks
8 Weeks
S11
S11-V1
S11-V2
S11-V3
S13
S13-V1
S13-V2
S13-V3
S14
S14-V1
S14-V2
S14-V3
S15
S15-V1
S15-V2
S15-V3
S19
S19-V1
S19-V2
S19-V3

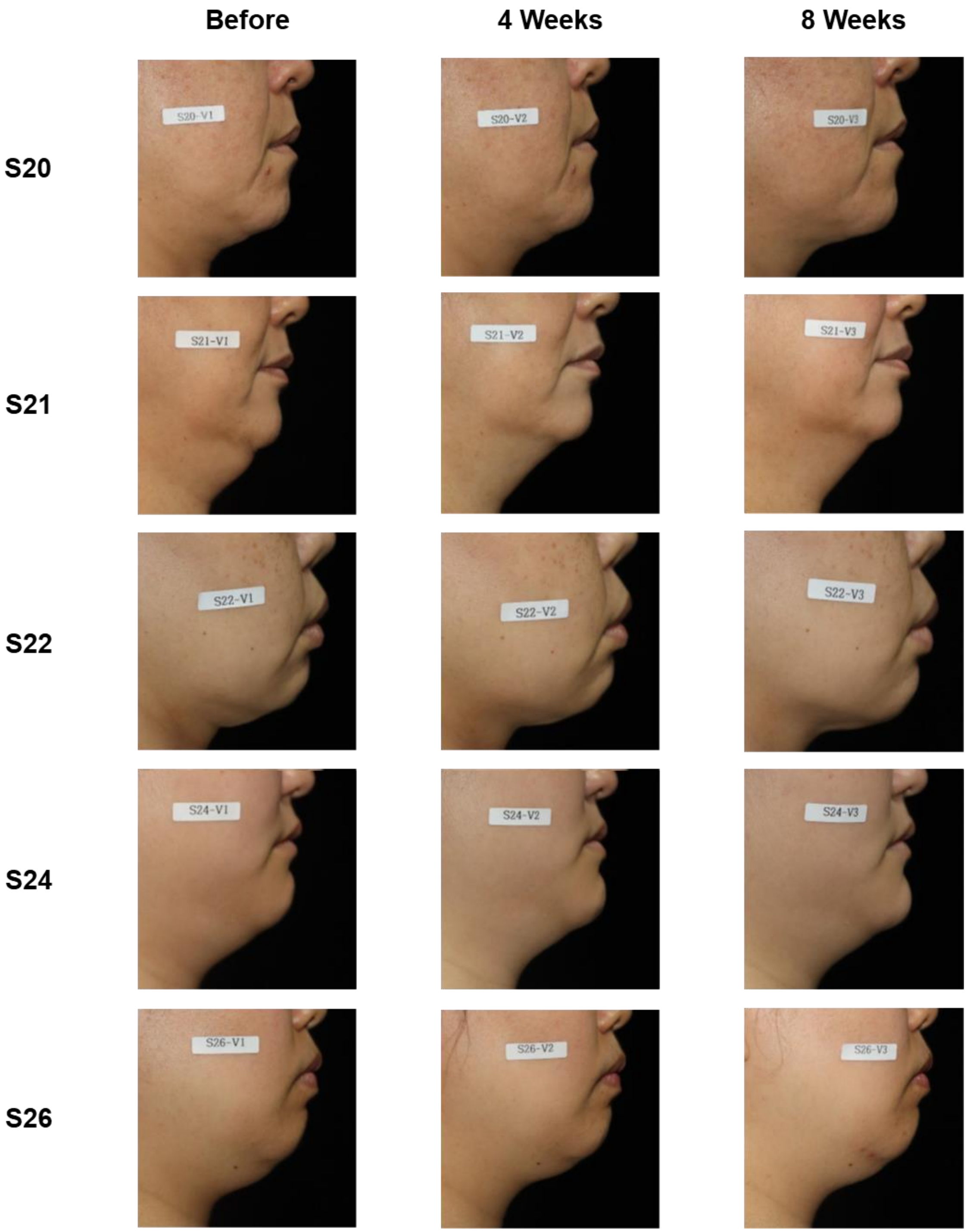
Before
4 Weeks
8 Weeks
S20
S21
S22
S24
S26

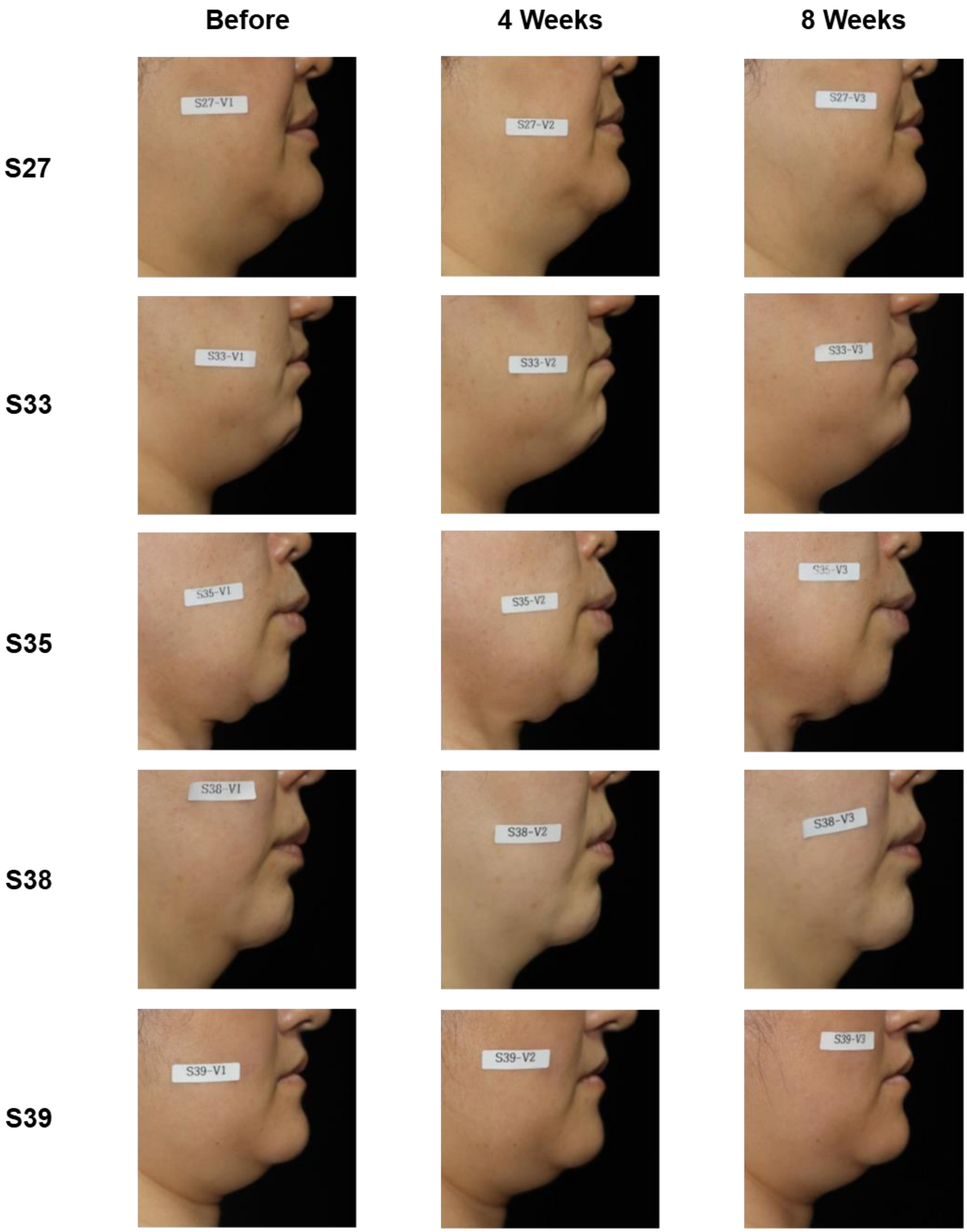
Before
4 Weeks
8 Weeks
S27
S33
S35
S38
S39

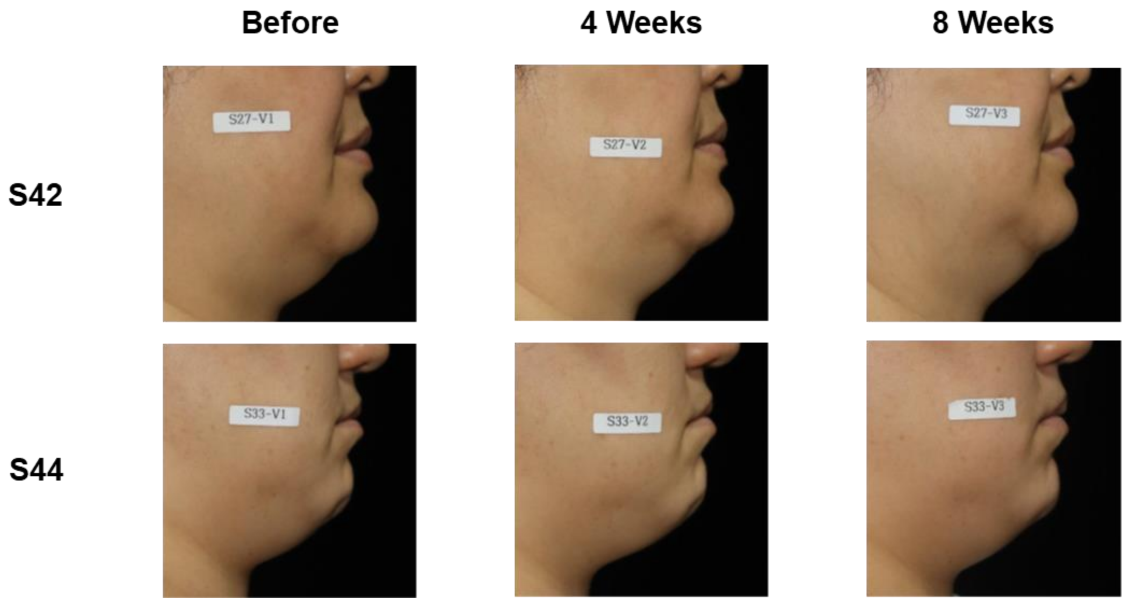

Figure S3. Clinical photographs of the submental fat evaluation through image analysis (DSLR).

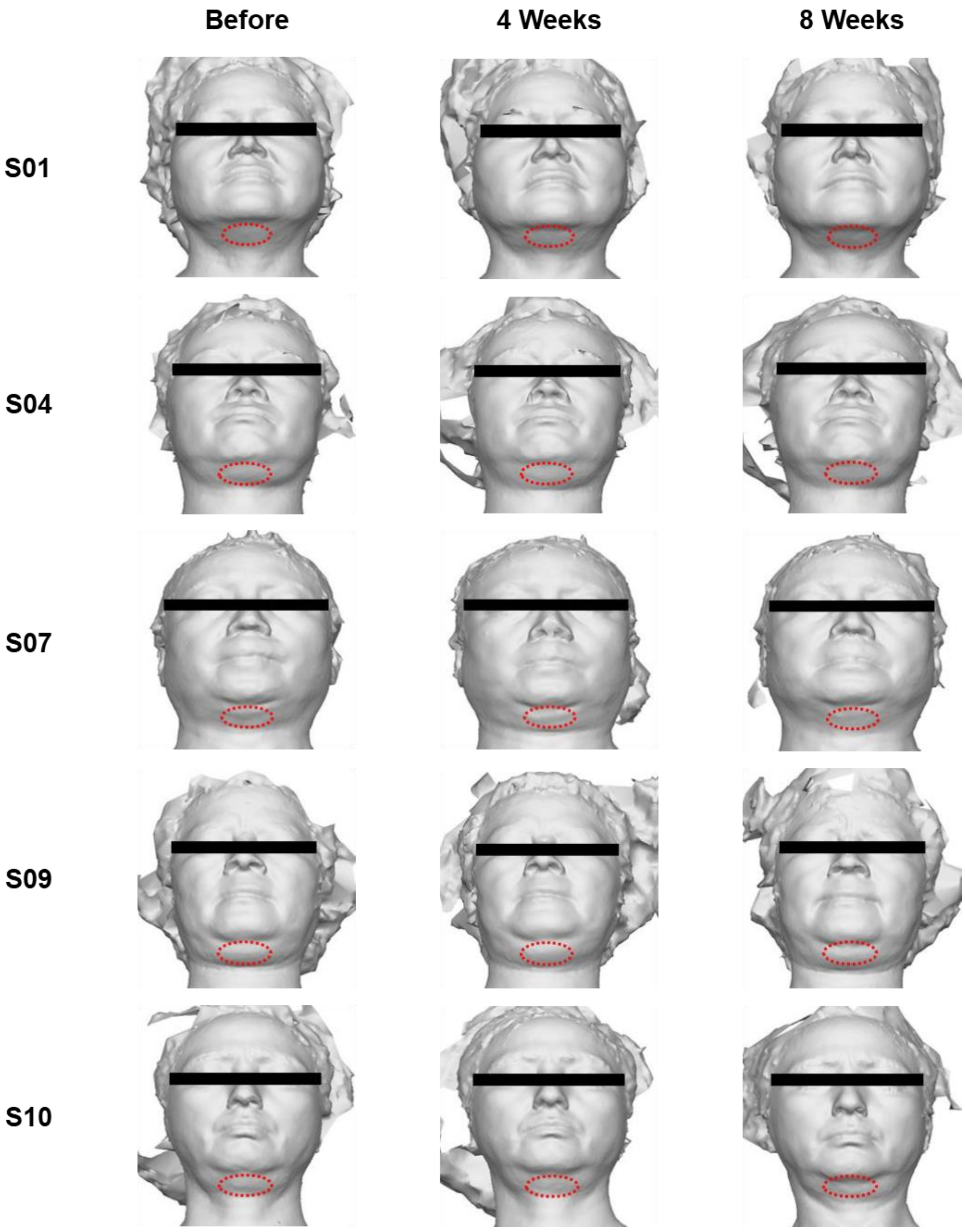
Before
4 Weeks
8 Weeks
S01
S04
S07
S09
S10

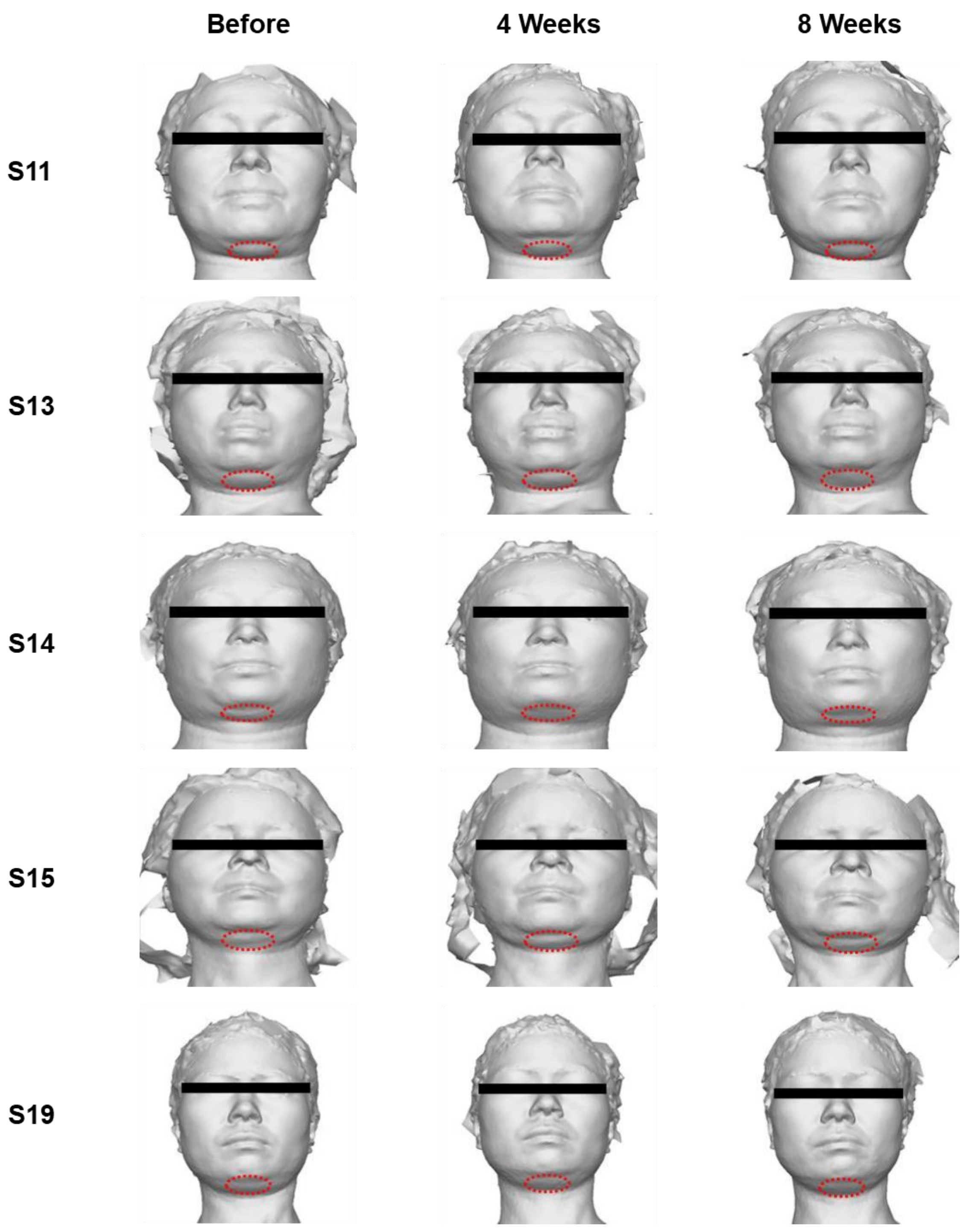
Before
4 Weeks
8 Weeks
S11
S13
S14
S15
S19

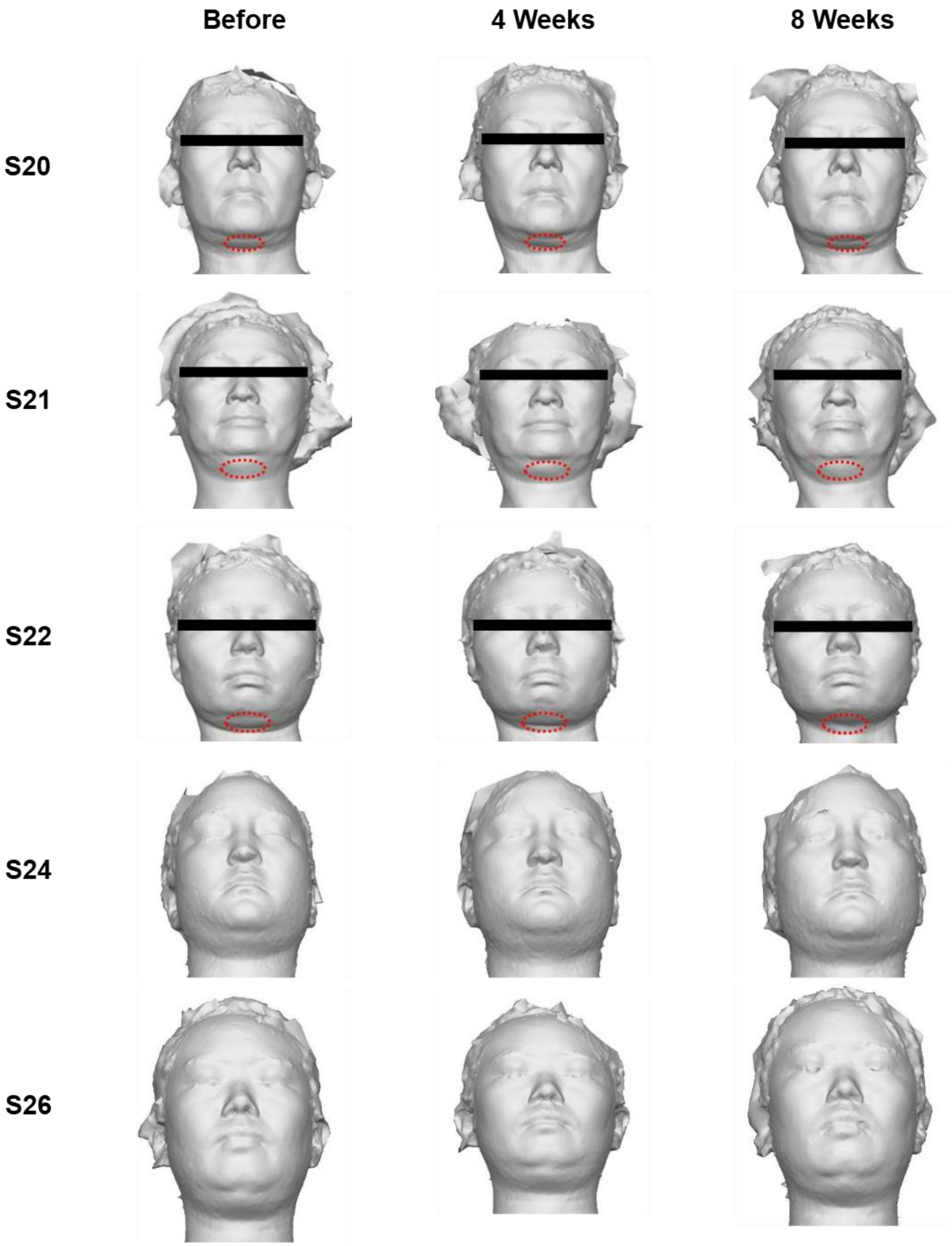
Before
4 Weeks
8 Weeks
S20
S21
S22
S24
S26

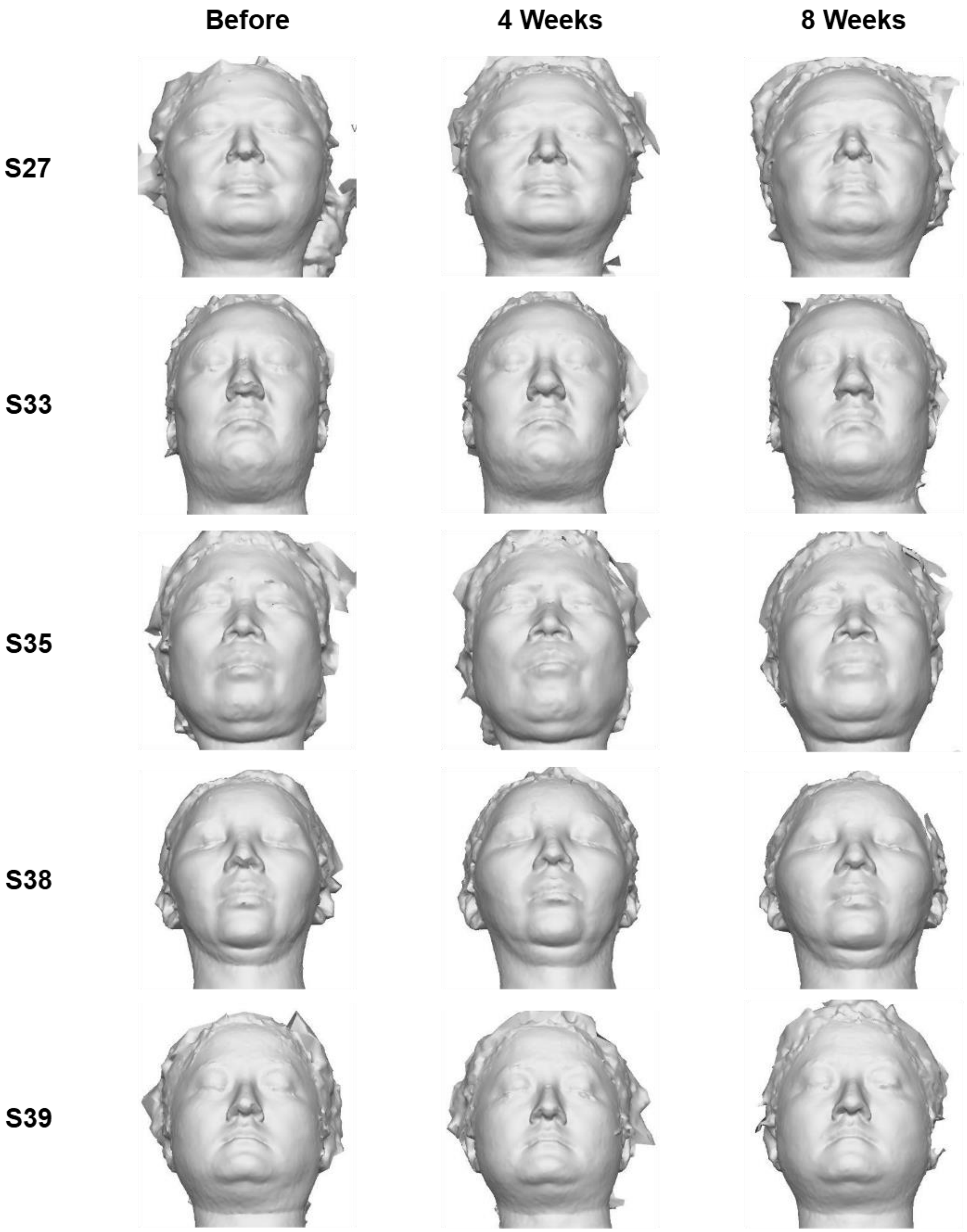
Before
4 Weeks
8 Weeks
S27
S33
S35
S38
S39

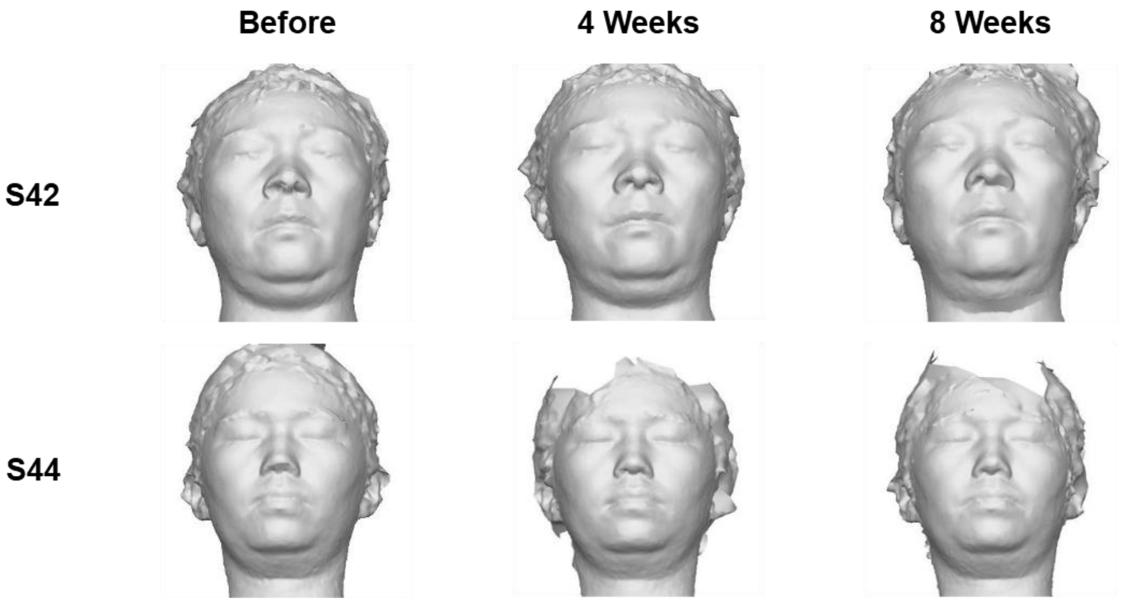

Figure S4. Clinical photographs of the submental fat evaluation through image analysis (Vectra XT).
